# A novel synthetic 3,4,5-tri-feruloylquinic acid enhances learning and memory via neurotrophin signaling in an aging model senescence-accelerated prone 8 mice

**DOI:** 10.1007/s11357-025-01783-7

**Published:** 2025-07-23

**Authors:** Hongyu Lin, Kazunori Sasaki, Farhana Ferdousi, Shinji Kondo, Hiroko Isoda

**Affiliations:** 1 Tsukuba Life Science Innovation (T-LSI) Program, University of Tsukuba, 305-8577 Tsukuba, Japan; 2 Open Innovation Laboratory for Food and Medicinal Resource Engineering (FoodMed-OIL), National Institute of Advanced Industrial Science and Technology (AIST), Tsukuba, 305-8577 Japan; 3 Alliance for Research on the Mediterranean and North Africa (ARENA), University of Tsukuba, Tsukuba, 305-8577 Japan; 4 Institute of Life and Environmental Sciences, University of Tsukuba, Tsukuba, 305-8572 Japan

**Keywords:** 3,4,5-Tri-feruloylquinic acid, SAMP8, Neurotrophin, Synaptic growth, Neurotransmitter, Neuroinflammation, BDNF

## Abstract

This study is the first to investigate the anti-aging effects of the newly synthesized compound, 3,4,5-tri-feruloylquinic acid (TFQA), in vivo using the senescence-accelerated mouse prone 8 (SAMP8) model, through integrated whole-transcriptomic and biochemical analyses. Oral administration of TFQA (1 mg/kg body weight) for 37 days led to significant cognitive improvements, as demonstrated by enhanced performance in the Morris water maze (MWM) test, including reduced escape latency and increased time spent in the target zone, target crossings, distance traveled, and swimming speed. Gene Ontology (GO) analysis of hippocampal microarray data revealed that TFQA upregulated genes associated with neurotransmitter and synaptic functions, while downregulating inflammatory pathways. Protein–protein interaction (PPI) analysis indicated that neurotrophin signaling was a critical upstream regulator in TFQA-treated mice. Further biochemical validation showed elevated levels of neurotransmitters (serotonin, noradrenaline, and dopamine) in the brain. Additionally, mRNA expression of tumor necrosis factor (*Tnf*), an inflammatory cytokine, was significantly reduced in the brain. Examination of the neurotrophins involved in neurotrophin signaling revealed that TFQA most prominently regulated brain-derived neurotrophic factor (*Bdnf*) co-expressed genes, which was validated by increased B*dnf* expression in both the brain and serum of SAMP8 mice. Bioluminescence imaging demonstrated that a single oral dose of TFQA significantly increased BDNF expression in the brains of BDNF-IRES-AkaLuc mice. Binding studies showed that TFQA has a strong affinity for the tyrosine receptor kinase B (TrkB) receptor. In summary, TFQA holds potential as a neuroprotective agent, mitigating learning and memory deficits in aging mice via neurotrophin signaling, particularly through BDNF upregulation.

**Graphical Abstract:**

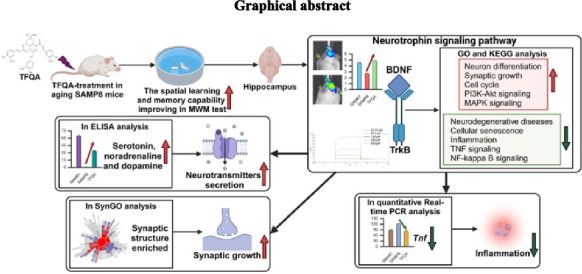

**Supplementary Information:**

The online version contains supplementary material available at 10.1007/s11357-025-01783-7.

## Introduction

Aging is widely recognized as the gradual decline that occurs over time in most organisms. It is a progressive and irreversible pathophysiological process. This process leads to a gradual and permanent decline in bodily functions across all organ systems, reduces tissue and cellular function, and significantly increases the risk of age-related diseases. These diseases include neurodegenerative, cardiovascular, metabolic, musculoskeletal, and immune system disorders. The underlying cause is the accumulation of damage due to various stressors [1]. Genomic instability, telomere attrition, epigenetic alterations, lack of protein homeostasis, dysregulated nutrient sensing, mitochondrial dysfunction, cell senescence, stem cell exhaustion, and altered intercellular communication are hallmarks of aging [2]. During normal aging, gradual and process-specific declines occur in various perceptual, cognitive, and action-related processes. Older adults typically perform worse than younger adults on response latency and accuracy in a variety of tasks, including perceptual speed, working memory, tracking, decision-making, episodic memory, and multitasking [3]. In the brain, aging causes many changes and leads to a progressive decline in cognitive abilities. Typically, aging-related brain changes include tissue atrophy, altered neurotransmitter levels, and the accumulation of damage in the cellular environment. Structural changes trigger disruption of the peripheral neurofibrillary network, leading to cognitive impairment and dementia. Aging is also associated with abnormal neuronal function [4]. Physiological brain aging is characterized by a loss of neuronal apoptosis and synaptic connections, which results in age-related declines in sensory processing, motor performance, and cognitive function [5]. The aging brain is vulnerable to oxidative stress, which is a key cause of age-related neurodegeneration. Neuroinflammation is a major component of the early pathogenesis of Alzheimer’s disease (AD) and may be causally involved in selective neuronal death [6]. Research on these topics helps design and implement interventions aimed at improving the retention of cognitive function in late life.

Recently, neurotrophic factors have been shown to play a critical role in brain aging. The main function of these proteins is to regulate axonal growth and neuronal differentiation. The neurotrophin family includes nerve growth factor, neurotrophin-3, neurotrophin-4/5, and, most importantly, brain-derived neurotrophic factor (BDNF) [7]. BDNF is the most abundant neurotrophic factor in the brain and is crucial for the survival of neurons during development and the formation of adult brain neural networks. It exerts its biological effects through tyrosine receptor kinase B (TrkB) [8]. Higher levels of BDNF expression in the brain are associated with slower cognitive decline and may also reduce the harmful effects of neurodegenerative diseases on cognitive function [9]. Dysregulated BDNF/TrkB signaling leads to dendritic spine loss and apoptosis in aging mice, resulting in cognitive impairments [10]. Therefore, drug-induced increases in BDNF levels have emerged as a therapeutic approach with great potential.

**Fig. 1:**
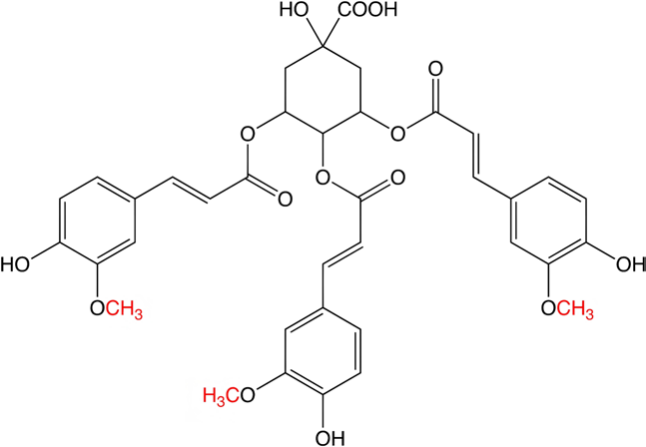
The structure of TFQA

Quinic acid (QA) is a cyclohexanecarboxylic acid found in the extracts of various plant parts, including coffee beans, sweet potatoes, apples, and peaches. QA improves inflammation by inhibiting the NF-κB signaling pathway and the MAP kinase pathway regulated by tumor necrosis factor (TNF) [11]. QA and its derivatives from *Gardenia jasminoides* have antioxidant activity by inhibiting lipid peroxidation [12]. QA induces cell cycle arrest at the G0/G1 phase and acts on actin cytoskeleton organization, chromatin remodeling, neuronal differentiation, and bone morphogenetic protein signaling [13]. Purple sweet potato extracts enriched with QA have neuroprotective effects on the brain in vivo by improving spatial learning and memory capabilities [14]. QA can promote neurite outgrowth and maintain cell viability in amyloid Aβ-induced PC12 cells, thereby playing a neuroprotective role in AD. QA and its derivatives can induce neuronal cell differentiation and upregulate neurotrophic factors such as nerve growth factor (NGF) and BDNF [15]. Among the QAs, feruloylquinic acid (FQA) can penetrate the brain to protect the blood–brain barrier and brain structure. It effectively induces the expression of heme oxygenase-1 protein in cultured astrocytes, exerting a direct positive effect on neurons and glial cells to improve neurological dysfunction [16]. The FQA-enriched extract was found to enhance antioxidant activity and protect against oxidative stress, as FQA may inhibit H_2_O_2_-induced extracellular signal-regulated kinase 1/2 (ERK1/2) activation, thereby attenuating cell apoptosis and enhancing neuroprotection [17]. As one of the FQAs, the new synthetic substance 3,4,5-tri-feruloylquinic acid (TFQA) has been shown to have a strong neural effect on improving neurogenesis in neural stem cells from adult mouse brains, as demonstrated in our previous paper (Fig. 1) [18].

Therefore, in this study, we researched the neural and anti-aging effects of TFQA in vivo in aging mice and discussed its molecular mechanism in treating aging through genomics and protein content analysis. We also provide insights into the significance of TFQA as a potential anti-aging drug and its preventive effects on neurodegenerative diseases.

## Material and methods

### Sample preparation

TFQA was provided by the National Institute of Advanced Industrial Science and Technology (AIST) and dissolved in 70% ethanol for preparation.

### Animal grouping and treatment

Six-month-old adult male senescence-accelerated mouse prone 8 (SAMP8) and senescence-accelerated mouse resistant 1 (SAMR1) (SLC company, Japan) mice were used (purchase number: 23–393). All mice were allowed to acclimate to laboratory conditions for 7 days and were kept in a clean room maintained at a controlled temperature of 25 ± 2 °C with an automatically controlled light–dark cycle (12 h light, 12 h dark) and free access to food and water. SAMR1 mice (*n* = 9) were used as normal aging controls. The SAMP8 control group was treated with water (*n* = 9), while the SAMP8 treatment group was administered with TFQA (*n* = 9). TFQA (1 mg/kg) was added to drinking water and administered once daily by oral gavage for 37 days [14]. In our previous study, TFQA demonstrated effectiveness in promoting neural stem cell activity, as indicated by an increase in neurosphere size, even at a relatively low concentration of 1 µM [18]. Based on these results, we selected 1 mg/kg as the dose for the in vivo study, considering both the compound’s previously observed efficacy at low concentrations and the typical low-dose range commonly used in studies involving polyphenolic compounds. This dose was expected to exert biological activity while minimizing potential toxicity.

On the 38th day, mice were anesthetized with carbon dioxide and dissected, and organs were collected. All procedures were approved by the Gene Research Center and conducted in accordance with the guidelines for the Care and Use of Animals, approved by the Council of the Physiological Society of Japan.

### Morris water maze (MWM) test

The MWM test was accomplished using a circular pool with a diameter of 120 cm and a height of 45 cm, featuring a featureless inner surface. The pool was filled to a depth of 30 cm with water at 25 ± 2 °C and divided into four quadrants. A platform (10 cm in diameter) was placed in the northeast quadrant and submerged 1 cm below the water surface, making it invisible at water level. Mice were given four trial sessions each day for 7 days, with the platform fixed in the same location throughout the experiment. Each mouse was allowed to remain on the platform for 15 s every day for 7 days.

After 7 days of trials, the probe test was performed and the hidden platform was removed from the pool, and the mice were placed in the opposite location of the platform and were allowed to swim for 60 s. The number of crossings over the previous location of the platform, which was hidden during the acquisition trials, was recorded as a measure of spatial memory.

### RNA and protein isolation

All of the mice were euthanized by cervical dislocation. The whole brain of each mouse was quickly removed. The cerebral cortex and hippocampus were dissected and rapidly frozen in liquid nitrogen. Serum samples were collected from the femoral artery and were incubated at 4 °C for 24 h and then centrifuged at 1000 × g for 30 min. The serum was collected and stored immediately at − 20 °C until use. Total RNA was extracted using the ISOGEN kit (Nippon Gene, Japan). The quality and quantity of the RNA were assessed with the NanoDrop One/OneC (Thermo Fisher Scientific, USA). Proteins from cerebral cortex and hippocampus were isolated in 1 mL of radioimmunoprecipitation assay (RIPA) buffer with a protease inhibitor (Santa Cruz Biotechnology, Inc., Tokyo, Japan). The supernatant was kept for protein analysis.

### DNA microarray analysis

We performed microarray analysis starting with 100 ng RNA per sample utilizing the GeneChip® Whole Transcript (WT) PLUS microarray reagents and kits (ThermoFisher Scientific, Waltham, MA, USA) in accordance with the manufacturer’s recommended protocols. For cDNA synthesis, 5 µL of RNA was mixed with First-Strand Buffer and Enzyme and incubated at 25 °C for 1 h, followed by second-strand synthesis using Second-Strand Mix with incubation at 40 °C for 16 h and subsequent cooling to 4 °C. Single-stranded cDNA was generated by incubating 15 µg cDNA with 2nd-Cycle ss-cDNA Master Mix, followed by fragmentation using Fragment Master Mix to produce fragmented ss-cDNA samples. The samples were then hybridized to GeneChip Clariom S arrays (Mouse; ThermoFisher Scientific, Waltham, MA, USA), washed, and scanned using the GeneAtlas Imaging Station. Following acquiring raw image data from the scanning process, subsequent analysis was conducted utilizing the Transcriptome Analysis Console (TAC) software, version 4.0.2 (ThermoFisher Scientific, Waltham, MA, USA). The raw data were normalized to ensure robustness using the signal space transformation robust multi-chip analysis (SST–RMA) algorithm. A one-way ANOVA was applied to identify the differentially expressed genes (DEGs). Additional bioinformatics analyses were conducted using Database for Annotation, Visualization, and Integrated Discovery (DAVID), Metascape, Bioinformatics, Morpheus, NetworkAnalyst, Enrichr, and Synaptic Gene Ontologies (SynGO). The complete microarray dataset has been deposited in Gene Expression Omnibus (GEO) under accession number GSE283354.

### Enzyme-linked immuno-sorbent assay (ELISA)

The ELISA kits were used to measure dopamine (Ref: BA-E-5300R, ImmuSmol, Australia), serotonin (Ref: BA-E-5900R, ImmuSmol, Australia), and noradrenaline (Ref: BA-E-5200R, ImmuSmol, Australia) contents in the cerebral cortex of mice brains. The ELISA kit (Catalogue Number: KE00096, Proteintech Group Inc., USA) was used to measure BDNF in the serum, cerebral cortex, and hippocampus.

### Biacore assay

Preparation of TrkB-immobilized sensor chips: The extracellular domain of human recombinant TrkB protein (10,047-H02H, Sino Biological Inc., China) was reconstituted in distilled water to a concentration of 0.25 mg/mL. It was then diluted to 50 µg/mL using 10 mM sodium acetate (Cytiva, Japan). Immobilization was performed using an amine coupling kit containing 1-ethyl-3-(3-dimethylaminopropyl) carbodiimide hydrochloride, N-hydroxysuccinimide, and 1 M ethanolamine hydrochloride-NaOH (pH 8.5) (Cytiva, Japan). The analyte solution (500 µM) was further diluted in HBS-EP + buffer containing 0.5% (v/v) dimethyl sulfoxide to concentrations of 31.25, 67.5, 125, 250, and 500 µM. The analyte solutions were injected into the TrkB ECD-immobilized sensor chip set in the Biacore X100 system (Cytiva, Japan) at a flow rate of 30 µL/min (contact time, 120 s; dissociation time, 200 s). Biacore Insight evaluation software was employed to analyze the resonance units (RU), equilibrium constant (*K*_*D*_ in M), association rate constant (*Ka* in M^−1^ s^−1^), and dissociation rate constant (*K*_*d*_ in s^−1^) after conducting the kinetics assay (1:1 binding). The theoretical maximal response (Rmax) in RU was calculated using the following formula:

Theoretical Rmax = $\frac{{\mathrm{a}}\text{nalyte molecular weight}}{{\mathrm{l}}\text{igand molecular weight}}$ RU valence of the ligand.

### BDNF-AkaLuc mice

The CRISPR-Cas9 ribonucleoprotein complex of *Bdnf* was complicated into zygotes of male C57BL/6 J mice (Jackson Laboratory, Japan). The modified zygotes were transferred into oviducts in pseudopregnant ICR females (Jackson Laboratory, Japan), and newborns were obtained (purchase number: 24–013).

### AkaLumine-AkaLuc bioluminescence imaging

To examine noninvasive BDNF expression in vivo, AkaLumine-AkaLuc bioluminescence imaging was performed in Bdnf-AkaLuc mice. AkaLumine (Fujifilm Wako, Japan) was administered intraperitoneally at a dosage of 10 mg/kg TFQA. The AkaLumine solution was prepared on ice. Images were captured using the In Vivo Imaging System (IVIS) at a rate of one image/min, and luminescence intensity was monitored over time. The TFQA dose used in SAMP8 mice was 1 mg/kg, whereas a higher dose of 10 mg/kg was administered in BDNF-AkaLuc reporter mice. This difference reflects the distinct purposes of each experiment: in SAMP8 mice, a lower dose was sufficient to assess behavioral, transcriptomic, and molecular outcomes associated with aging and neurodegeneration. In contrast, a higher dose was employed in BDNF-AkaLuc mice to enhance the detection sensitivity of bioluminescence imaging and to ensure a robust transcriptional response of the BDNF promoter, which was necessary for reliable visualization of signal changes in vivo.

#### SH-SY5Y cell culture

The human neuroblastoma cell line SH-SY5Y was obtained from the American Type Culture Collection (ATCC, USA). The cells were cultured in a 1:1 (v/v) mixture of Dulbecco’s modified Eagle’s medium (DMEM) and Ham’s F-12 Medium (Gibco, USA), supplemented with 15% fetal bovine serum (FBS) (FUJIFILM Wako, Japan), MEM nonessential amino acids (FUJIFILM Wako, Japan), and 1% penicillin (5000 µg/mL)-streptomycin (5000 IU/mL) solution (Sigma-Aldrich, USA) as the growth medium in a 75-cm^2^ flask. The flasks were maintained in an atmosphere of 5% CO_2_/95% humidified air at 37 °C.

#### SH-SY5Y cell treatment

TFQA was dissolved and diluted in serum-free Eagle’s Minimum Essential Medium (OPTI-MEM, Gibco, USA) to achieve treatment concentrations ranging from 10 to 40 µM. The control group was prepared without TFQA treatment. After 24 h of seeding, the SH-SY5Y cells of P11 were cultured in OPTI-MEM for an additional 24 h. Subsequently, they were incubated for 24 h for further analysis.

In addition, an inhibitor experiment was performed to analyze the target of TFQA in the TrkB/BDNF pathway. The cells were treated with 10 and 20 µM TFQA and co-treated with the inhibitor, 10 µM ANA-12 (Selleck Biotech, Japan), as a TrkB antagonist, for an additional 24 h.

#### 3-(4,5-Dimethylthiazol-2-yl)−2,5-diphenyltetrazolium bromide (MTT) assay

Cell viability activity was determined using the MTT assay to check the cytotoxicity of TFQA. SH-SY5Y cells were seeded at 2 × 10^5^ cells/mL in 96-well plates (BD BioCoat, USA) and incubated for 24 h. The cells were then treated with 10–40 µM TFQA for 24 and 48 h, respectively. MTT solution (DOJINDO, Japan) dissolved in PBS (Sigma-Aldrich, USA) was added (10 µL/well) and incubated for an additional 24 h. The resulting MTT formazan was dissolved in 100 µL of 10% sodium dodecyl sulfate (SDS) (w/v) (Nippon Gene, Japan), and absorbance was measured using a microtiter plate reader (Dainippon Sumitomo Pharma, Japan). The absorbance values were normalized to that of the culture medium, and viability was calculated as a percentage (%) of the untreated cells.

### Quantitative real-time PCR analysis

RNA samples were used for reverse transcription PCR with the SuperScript III Reverse Transcription Kit (Invitrogen, CA, USA). In RNA samples of SAMP8 and SAMR1 mice, *Gapdh* (Mm99999915_g1) (Applied Biosystems, CA, USA) was considered control. *Tnf* (Mm00443258_m1) and *Bdnf* (Mm04230607_s1) were selected for analysis using the quantitative Real-Time PCR Software 1.3.1 (Applied Biosystems, CA, USA). In RNA samples from SH-SY5Y cells, *GAPDH* (Hs02786624_g1) was used as the control, and *BDNF* (Hs02718934_s1) was selected for analysis using the quantitative Real-Time PCR Software 1.3.1.

### Statistical analysis

The results are presented as the mean ± standard deviation (SD). Statistical analysis was performed using GraphPad Prism 6 (GraphPad Software, USA). The data were analyzed using ordinary one-way ANOVA followed by Tukey’s multiple comparisons test to assess differences between groups. The significance threshold was set at 0.05, with adjusted *P*-values reported for multiple comparisons.

## Results

### TFQA improved spatial memory and learning capabilities in aging mice

**Fig. 2:**
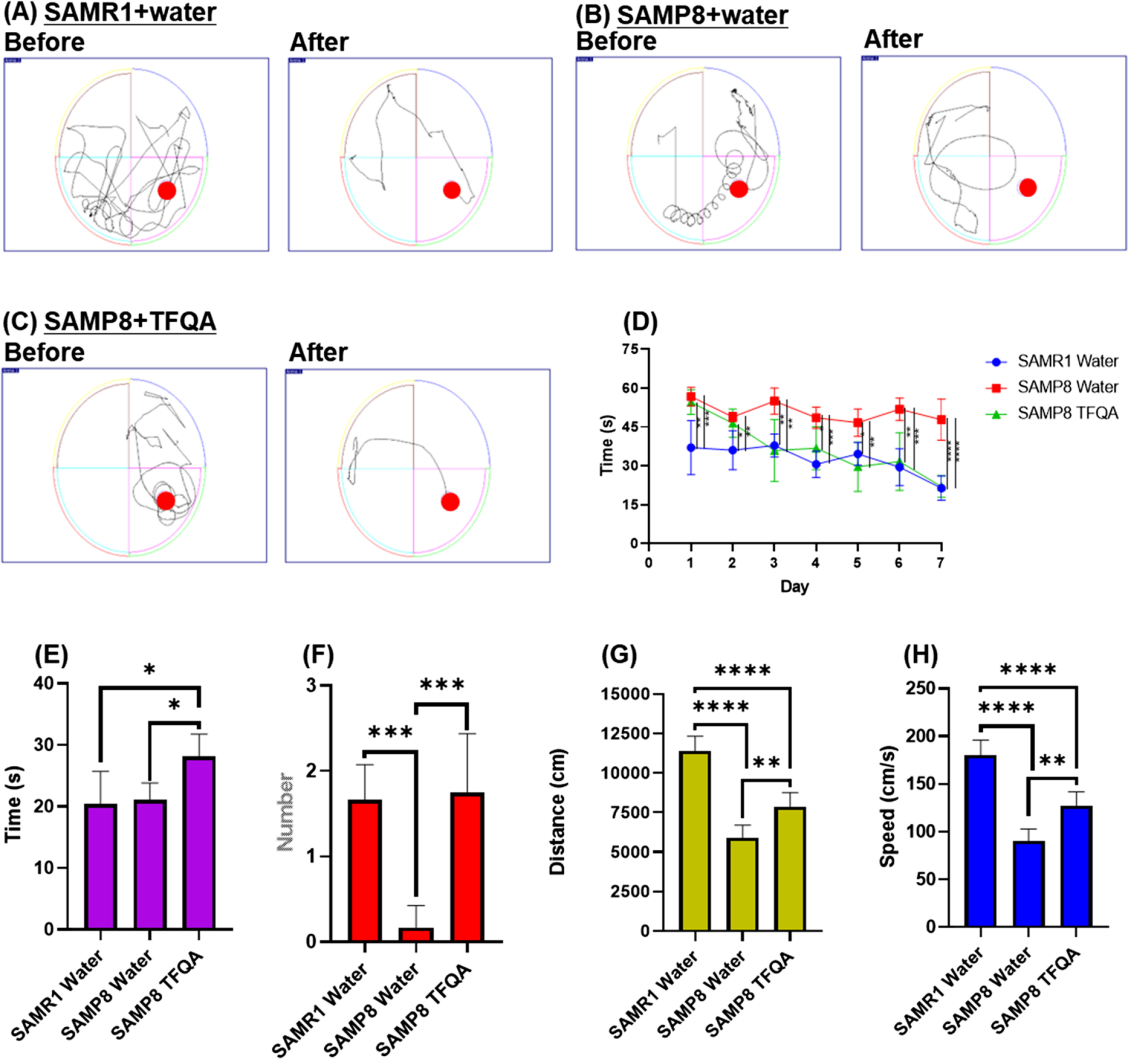
Behavioral test results from Morris water maze in treatment groups. Swimming trajectories during training trials are shown for **A** SAMR1 + water, **B** SAMP8 + water, and **C** SAMP8 + TFQA (filled circles: the start point and platform). Quantitative assessments include **D** escape latency, **E** time spent in the target quadrant, **F** number of platform crossings, **G** swimming distance, and **H** swimming speed during the probe test (*n* = 6 in each group). Data are expressed as the mean ± SD of three independent experiments. ****p < 0.0001, ***p < 0.001, **p < 0.01, and *p < 0.05 by ANOVA followed by Tukey’s post hoc test

To evaluate the effect of TFQA on spatial learning and memory impairment, SAMP8 mice were orally administered 1 mg/kg of TFQA for 37 days and compared with age-matched SAMR1 mice and untreated SAMP8 mice, both of which received water only. Spatial learning and memory were evaluated using the MWM test, a widely used behavioral assay. Figure 2A, B, and C show the training trials of mice in each group. Using video tracking, the successive swims of each mouse were recorded. As shown in Fig. 2D and Table S1, escape latency refers to the amount of time it takes for a rodent to locate and swim to a hidden platform in a pool of water. The escape latency of the SAMP8 TFQA-administered group decreased from the first to the seventh day of training (day 1, 54.6 ± 6.8 s; day 2, 46.5 ± 8.9 s; day 3, 40.0 ± 8.9 s; day 4, 36.9 ± 9.8 s; day 5, 29.7 ± 16.9 s; day 6, 31.7 ± 11.4 s; day 7, 22.1 ± 6.9 s), showing a statistically significant reduction in escape latency compared to the SAMP8 water-administered group. As expected, the SAMR1 water-administered group also showed a significant reduction in escape latency from day 1 of the trial session (day 1, 42.7 ± 17.3 s; day 2, 39.3 ± 11.9 s; day 3, 32.7 ± 8.3 s; day 4, 31.5 ± 6.6 s; day 5, 32.4 ± 5.3 s; day 6, 28.8 ± 8.4 s; day 7, 22.0 ± 6.3 s) compared to the SAMP8 water-administered group.

Moreover, to determine whether the effect of spatial learning and memory improvement through TFQA administration was sustainable, and whether the learning and memory capacity of the mice depended on spatial information, the probe test was conducted. In the probe test, the behavior of the mice was observed under the same conditions, except that the platform was removed. The time spent in the target area refers to the amount of time a rodent spends in the quadrant of the pool where the hidden platform was located during the test phase. The number of target crossings represents how often a rodent crosses the area where the hidden platform was located during the training phase. TFQA-treated groups showed a significant increase in both the number of crossings over the virtual platform (TFQA-administered group, 1.75 ± 0.75) and the time spent in the quadrant (TFQA-administered group, 28.1 ± 4.7 s) where the platform was placed, compared to the SAMP8 water-administered group (number of crossings, 0.17 ± 0.17; time spent in quadrant, 21.0 ± 2.0 s) (Fig. 2E and F and Table S2). In Fig. 2G and H and Table S2, distance represents the total distance traveled by the rodent in the water while searching for the hidden platform, and speed refers to the average swimming pace of the rodent while searching for the platform. The swimming distance significantly increased in TFQA-treated SAMP8 mice compared to water-treated SAMP8 mice, with distances of 7877.932 and 5890.603 cm, respectively. The swimming speed also improved significantly after TFQA treatment in the SAMP8 model. These results indicate that TFQA can effectively enhance spatial learning, cognition, and memory capacity in aging mice.

### TFQA regulated the brain transcriptome in aged mice

**Fig. 3:**
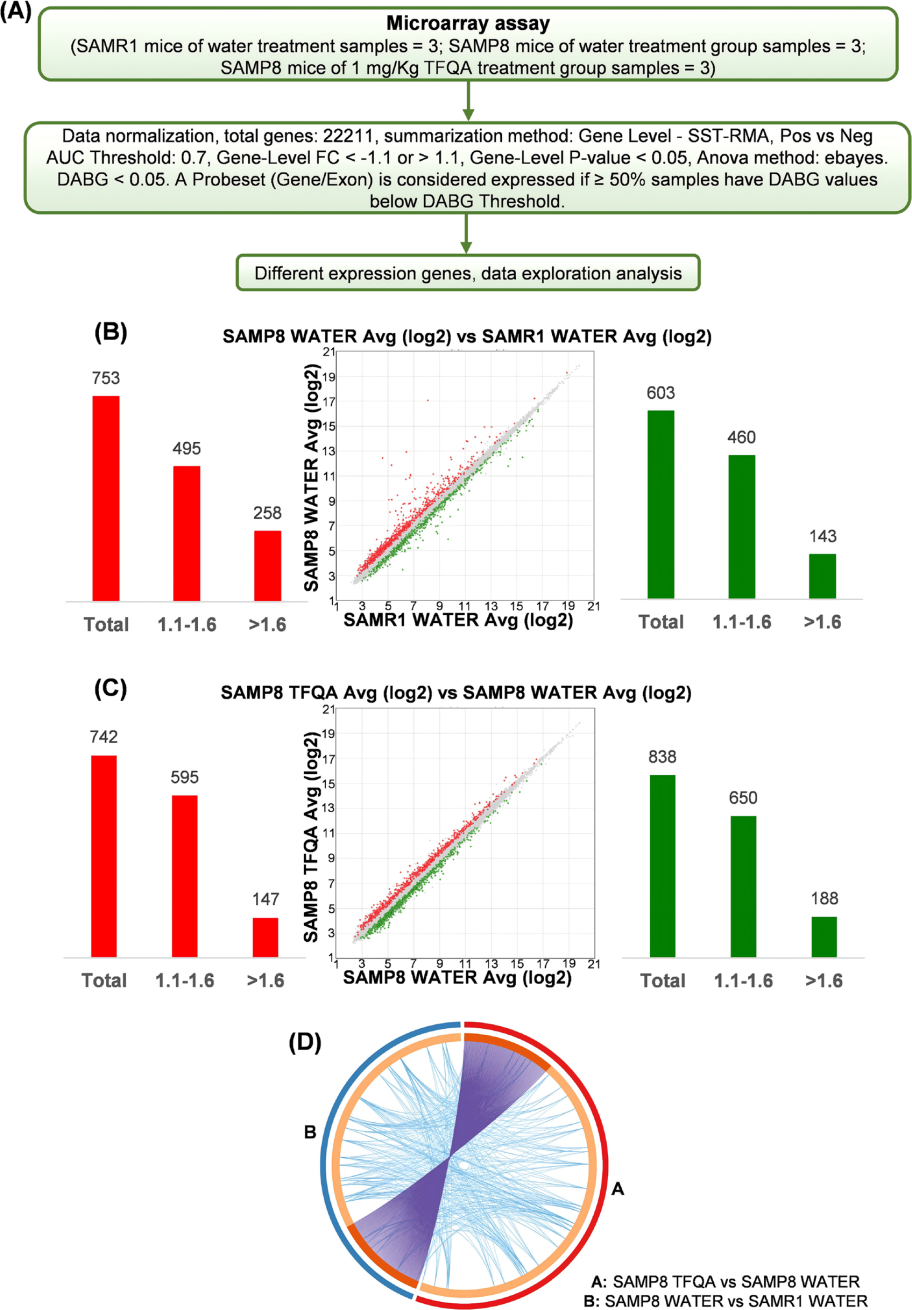
Overview of differentially expressed genes (DEGs) in the hippocampus of mice from different treatment groups. **A** The flowchart of microarray analysis. **B** DEGs between SAMP8 water vs. SAMR1 water groups. **C** DEGs between SAMP8 TFQA vs. SAMP8 water groups. Red represents upregulated DEGs and green represents downregulated DEGs. **D** Circos plot displaying the DEG overlap between SAMP8 water vs. SAMR1 water and SAMP8 TFQA vs. SAMP8 water comparisons. Each arc on the inner circle represents a gene list: dark orange indicates shared genes between conditions, and light orange indicates unique genes. Purple lines connect identical genes shared between groups, while blue lines link genes associated with the same gene ontology term or pathway. Significant DEG sets were identified based on *P*-values and fold changes (FC) using the one-way between-subject ANOVA in linear space, with thresholds set at *P*-value < 0.05 and FC > 1.1

To further explore the molecular mechanism of TFQA in treating aging, a microarray assay was performed on samples from the hippocampus. The hippocampus plays a key role in learning, memory formation, and consolidation [19]. The total microarray workflow is shown in Fig. 3A. Three hippocampal samples from SAMP8 mice treated with TFQA, three samples from SAMP8 mice treated with water, and three samples from SAMR1 mice treated with water were analyzed. Differentially expressed genes (DEGs) in the aging group compared with the SAMR1 group are expressed as SAMP8 water vs. SAMR1 water. DEGs in the TFQA treatment group compared with the aging group are expressed as SAMP8 TFQA vs. SAMP8 water. Figure 3B and C display the distribution of DEGs for each dataset, comparing the TFQA-treated and untreated SAMP8 mice groups and the untreated SAMP8 and SAMR1 mice groups (*P*-value < 0.05 and fold change (FC) > 1.1). The DEGs identified were 742 upregulated and 838 downregulated in SAMP8 TFQA vs. SAMP8 water. In SAMP8 water vs. SAMR1 water, 753 DEGs were upregulated and 603 were downregulated. In SAMP8 TFQA vs. SAMP8 water, 147 DEGs were upregulated and 188 were downregulated with the FC greater than 1.1, while in SAMP8 water vs. SAMR1 water, 258 DEGs were upregulated and 143 were downregulated with the FC greater than 1.1. Additionally, the circos plot in Fig. 3D illustrated the overlap of genes in the input genes list. Few common DEGs were found between SAMP8 water vs. SAMR1 water and SAMP8 TFQA vs. SAMP8 water. Together, these results suggest that TFQA may regulate DEGs to treat aging in SAMP8 mice.

### TFQA regulated neurotransmitter secretion, synaptic function, and neuroinflammation in mice hippocampus

**Fig. 4:**
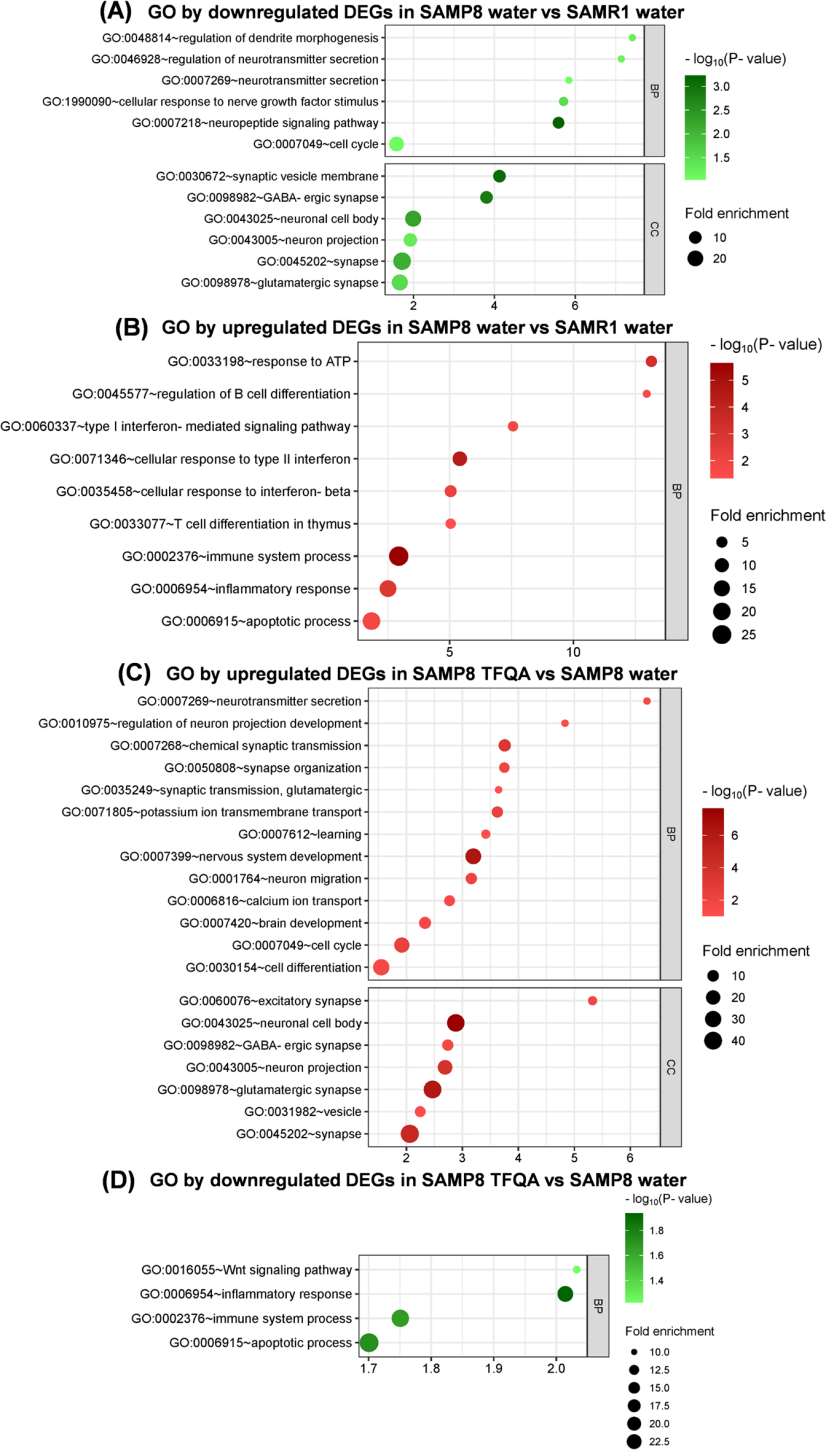
Biological events in the mice hippocampus. **A** Biological process (BP) and cellular component (CC) gene ontology (GO) enrichment of downregulated DEGs in SAMP8 water vs. SAMR1 water. **B** BP enrichment of upregulated DEGs in SAMP8 water vs. SAMR1 water. **C** BP and CC enrichment of upregulated DEGs in SAMP8 TFQA vs. SAMP8 water. **D** BP enrichment of downregulated DEGs in SAMP8 TFQA vs. SAMP8 water. Significant DEG sets were identified based on *P*-value and fold change (FC) using the one-way between-subject ANOVA in linear space, with significance thresholds set at *P* < 0.05 and FC > 1.1. Significantly overrepresented GO terms were identified based on hypergeometric distribution, with significance thresholds set at *P* < 0.05

To understand the regulated functions of TFQA in aging models, Gene Ontology (GO) analysis was performed to identify enriched biological processes (BP) and cellular components (CC). In the comparison of SAMP8 water vs. SAMR1 water (Fig. 4A and Table S3), significantly downregulated differentially expressed genes (DEGs) were enriched in several biological processes (BP) and cellular components (CC), including neurotransmitter secretion (GO:0007269), cell cycle regulation (GO:000749), neuronal cell body (GO:0043025), and synaptic function (GO:0045202). Figure 4B and Table S4 display BP analysis of upregulated DEGs in SAMP8 water vs. SAMR1 water, revealing significant enrichment in T cell differentiation in the thymus (GO:0033077), immune response (GO:0006955), inflammatory response (GO:0006954), and apoptotic processes (GO:0006915). Analysis of upregulated DEGs in SAMP8 TFQA vs. SAMP8 water (Fig. 4C and Table S5) showed significant enrichment in neural development-related processes, including neurotransmitter secretion (GO:0007269), synapse organization (GO:0050808), learning (GO:0007612), nervous system development (GO:0007399), brain development (GO:0007420), and cell cycle regulation (GO:000749). The CC analysis demonstrated that neuron growth and synapse formation were predominant after TFQA treatment, with significant terms including excitatory synapse (GO:0060076), neuronal cell body (GO:0043025), glutamatergic synapse (GO:0098978), and general synaptic function (GO:0045202). These findings indicate that neuronal and synaptic formation is impaired in aging SAMP8 mice compared to SAMR1 controls, and that TFQA treatment can modulate neural development, behavior, and cognitive functions. For downregulated DEGs in SAMP8 TFQA vs. SAMP8 water (Fig. 4D and Table S5), BP analysis revealed regulation of inflammatory functions, including the Wnt signaling pathway (GO:0016055), inflammatory response (GO:0006954), and immune system processes (GO:0002376). Notably, the wingless-related integration site/β-catenin (Wnt/β-catenin) pathway, which regulates β-catenin and promotes microglial inflammation, was regulated [20]. Interestingly, while apoptotic processes (GO:0006915) were significantly stimulated in SAMP8 water vs. SAMR1 water, they were downregulated following TFQA treatment. It is suggested that inflammatory functions are stimulated during aging, while TFQA administration alleviates this inflammation. Collectively, our findings demonstrate that TFQA treatment shows considerable potential to mitigate inflammation and enhance brain development by modulating neurotransmitter secretion, synaptic growth, and cognitive learning capabilities.

### TFQA mitigated neuroinflammation and promoted neural growth potentially via neurtrophin signaling

**Fig. 5:**
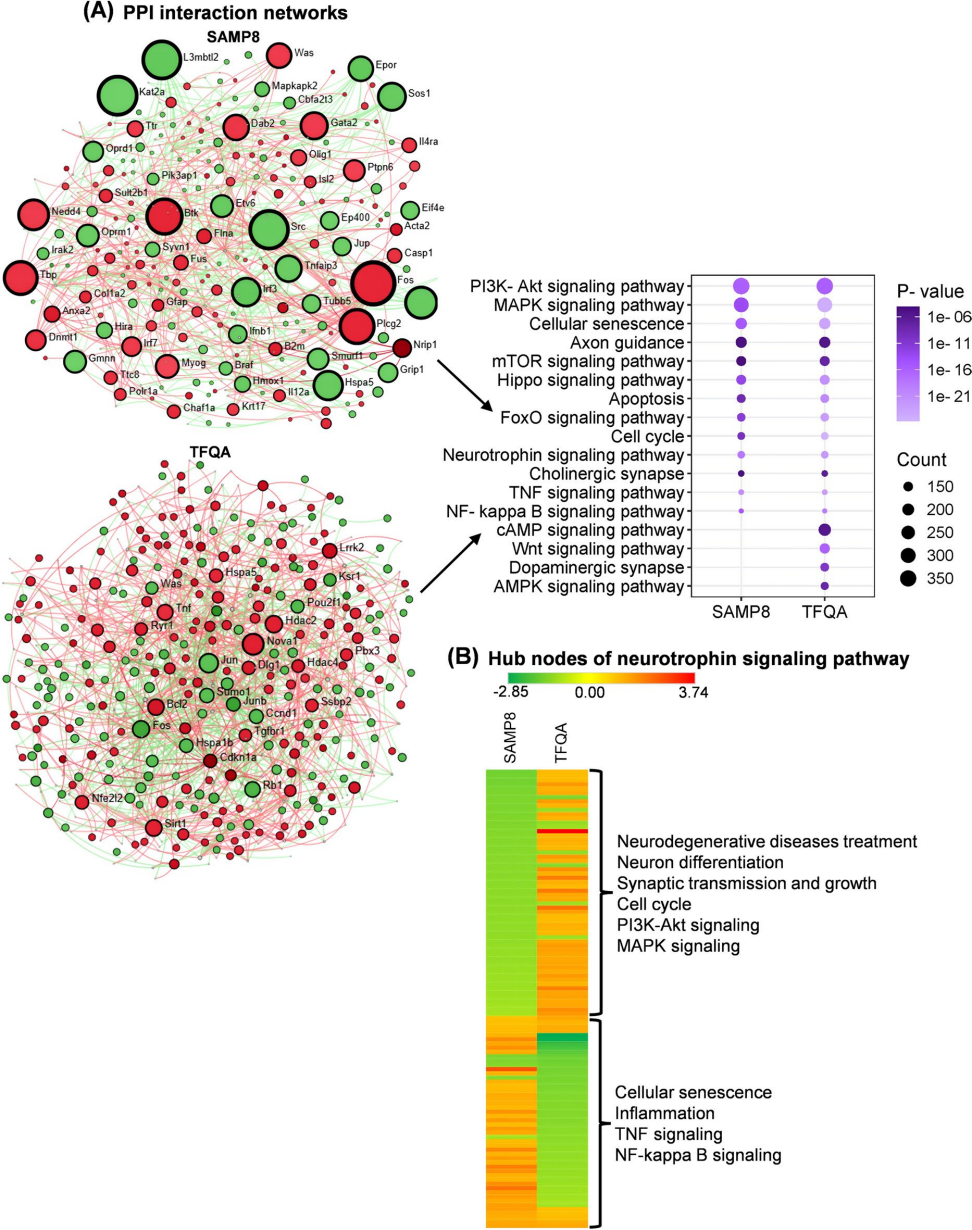
Protein–protein interaction (PPI) networks and enriched signaling pathways in the SAMP8 (SAMP8 water vs. SAMR1 water) and TFQA (SAMP8 TFQA vs. SAMP8 water) groups. **A** PPI networks by the DEGs and overrepresented KEGG pathways within the networks enriched in SAMP8 and TFQA groups. Significant DEG sets were identified based on *P*-value and fold change (FC) using the one-way between-subject ANOVA in linear space, with significance thresholds set at *P* < 0.05 and FC > 1.1. Significantly overrepresented KEGG pathways within the PPI networks were identified based on hypergeometric distribution, with significance thresholds set at *P* < 0.05. In the bubble plot, bubble color indicates the significance level (*P*-values) of KEGG pathway enrichment, while bubble size reflects the number of DEGs associated with each pathway. **B** The heatmap displays the FCs of hub nodes in the neurotrophin signaling pathway in the SAMP8 and TFQA groups. The color bar indicates FC values, with red representing upregulation and green representing downregulation. Overrepresented terms associated with the hub genes are shown on the right

To investigate the signaling pathways in SAMP8 water vs. SAMR1 water and SAMP8 TFQA vs. SAMP8 water, protein–protein interaction (PPI) and Kyoto Encyclopedia of Genes and Genomes (KEGG) analysis were performed. In the further analysis, SAMP8 refers to SAMP8 water vs. SAMR1 water, and TFQA refers to SAMP8 TFQA vs. SAMP8 water. The interaction PPI network of SAMP8 contained 931 nodes, 1169 edges, and 206 seeds. The interaction PPI network of TFQA included 1325 nodes, 1762 edges, and 292 seeds. In Fig. 5A, KEGG pathways enriched in SAMP8 and TFQA treatment were shown. These pathways include the phosphatidylinositol 3-kinase/protein kinase B (PI3K-Akt) signaling pathway, mitogen-activated protein kinase (MAPK) signaling pathway, cellular senescence, mammalian target of rapamycin (mTOR) signaling pathway, cell cycle, neurotrophin signaling pathway, TNF signaling pathway, nuclear component kappa B (NF-κB) signaling pathway, dopaminergic synapse, and MAPK signaling pathway. As an upstream signaling pathway, the neurotrophin signaling pathway regulates the MAPK and AKT and p53 and NF-κB signaling pathways to regulate growth, development, survival, and repair of the nervous system [21]. In Fig. 5B, the hub nodes heat map of the neurotrophin signaling pathway between SAMP8 and TFQA was shown. Hub nodes are the most interconnected proteins in the PPI network. The fold changes (FCs) of the hub nodes in the neurotrophin signaling pathway between SAMP8 and TFQA were significantly different. Upregulated hub nodes of TFQA were associated with the treatment of neurodegenerative diseases, neuron differentiation, synaptic transmission and growth, the cell cycle, PI3K-Akt signaling, and MAPK signaling. In the downregulated DEGs of TFQA compared to SAMP8, cellular senescence, inflammation, TNF signaling, and NF-κB signaling were identified. Therefore, in further analysis, the neurotrophin signaling pathway was focused on discussing. It is clarified that TFQA stimulates neurotrophin signaling to alleviate neuroinflammation and cell senescence while enhancing neural growth.

### TFQA enhanced synaptic structure via neurotrophin signaling pathway and improved neurotransmitter levels in the brains of aged mice

**Fig. 6:**
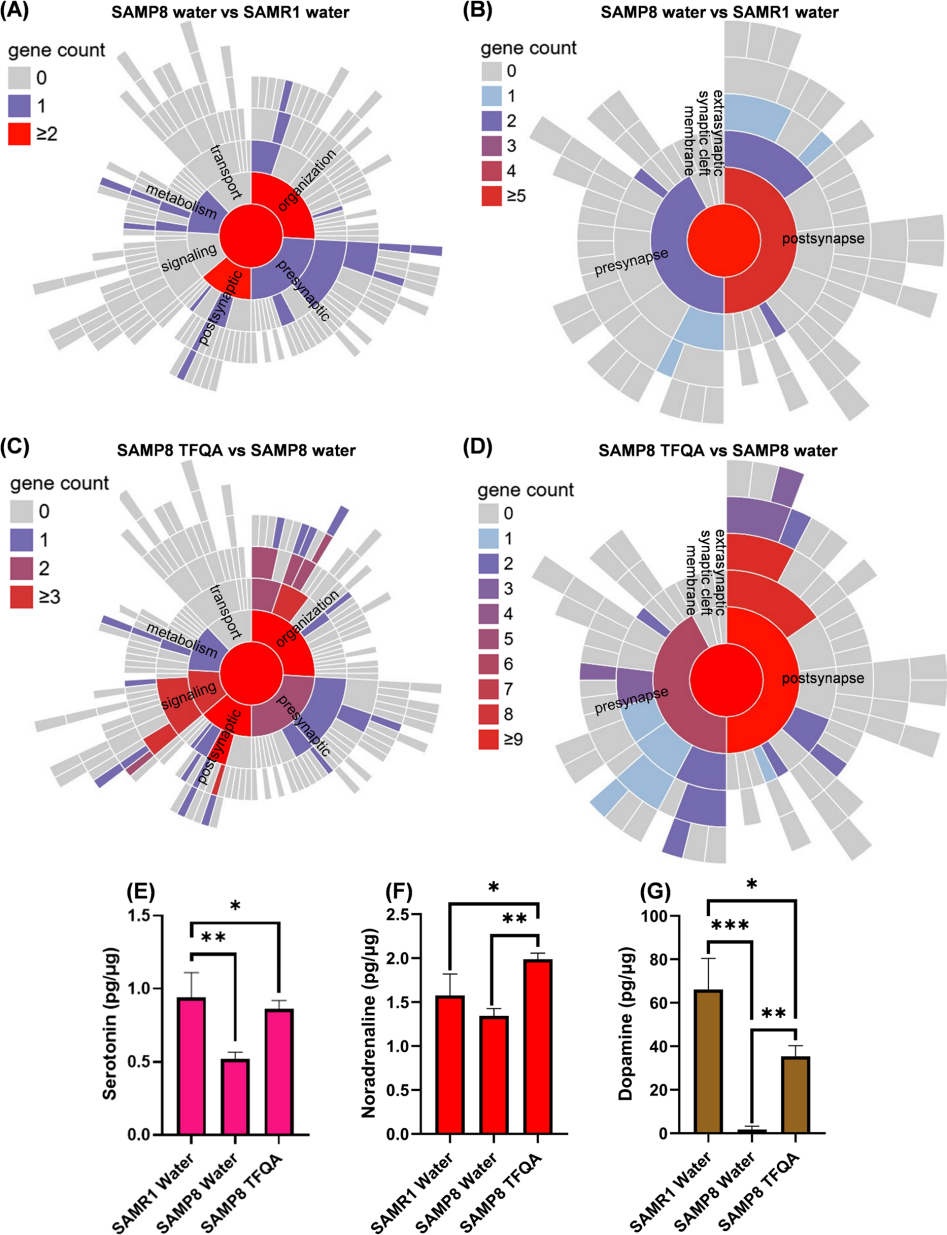
Synaptic function and neurotransmitter content in SAMP8 and SAMR1 mice following water or TFQA treatment. The sunburst plots displaying gene counts curated from the hub nodes of the neurotrophin signaling pathway that are associated synaptic genes based on SynGO annotations (https://www.syngoportal.org/). In SAMP8 group (SAMP8 water vs. SAMR1 water), gene counts are shown for **A** biological processes (BP) and **B** cellular components (CC). In TFQA group (SAMP8 TFQA vs. SAMP8 water), gene counts are shown for **C** BP and **D** CC. **E** Serotonin, **F** noradrenaline, and **G** dopamine contents in the cerebral cortex of SAMP8 and SAMR1 mice with TFQA and water treatments (*n* = 3 in each group). Data are expressed as the mean ± SD of three independent experiments. ****P < 0.0001, ***P < 0.001, **P < 0.01, and *P < 0.05 by ANOVA followed by Tukey’s post hoc test

To discuss the effect on improving the neurotrophin signaling pathway to enhance synaptic function, we performed SynGO analysis on the hub node lists of the neurotrophin signaling pathway in SAMP8 and TFQA. In Fig. 6A and Table S7 for SAMP8, processes in the synapse and synapse organization were significantly expressed in BP. In CC, as shown in Fig. 6B and Table S8, postsynapse was enriched. From the SynGO analysis of the neurotrophin signaling pathway hub nodes of TFQA, significant BP processes included postsynapse, regulation of postsynaptic membrane neurotransmitter receptor levels, processes in the synapse, neurotransmitter receptor localization to postsynaptic specialization membranes, synapse organization, synapse assembly, and chemical synaptic transmission (Fig. 6C and Table S9). In CC, as shown in Fig. 6D and Table S10, the hub nodes of the neurotrophin signaling pathway in TFQA regulated postsynaptic density, postsynapse, synapse, integral components of postsynaptic density membranes, integral components of presynaptic membranes, and presynapse. Specifically, TFQA remarkably enhanced postsynaptic function. It is demonstrated that in aging mice, TFQA induces the neurotrophin signaling pathway to improve synaptic growth function, thereby achieving the anti-aging effect.

Enhanced synaptic growth and plasticity influence action potentials and stimulate calcium channels, thereby enhancing neurotransmitter release [22]. The cerebral cortex critically supports memory, from short-term (seconds-minutes) to recent (1–2 days) and remote retention, as evidenced by inactivation and lesion studies. Its essential role encompasses consolidation, retrieval, and maintenance of memories, with demonstrated necessity in tasks ranging from fear recall to reward-place associations (Euston et al*.*, 2012). Due to the improvement in synaptic organization, we performed the ELISA assay to explore the effects of TFQA on neurotransmitters in the cerebral cortex. Serotonin levels in the cerebral cortex were assessed (Fig. 6E). Serotonin levels significantly increased after TFQA treatment, with a value of 0.86 pg/µg, compared to 0.52 pg/µg in the aging model. Figure 6F shows that enhanced noradrenergic activity during cognitively stimulating activities can delay the onset of behavioral and cognitive symptoms of neurodegenerative diseases. Compared to the SAMP8 and SAMR1 mice in the water treatment groups, noradrenaline content significantly increased, reaching 2.0 pg/µg in the SAMP8 mice treated with TFQA. In Fig. 6G, dopamine also increased in the TFQA-treated SAMP8 mice. Dopamine level was 35.3 pg/µg in the TFQA-treated SAMP8 mice, compared to 1.8 pg/µg in the aging mice. It is suggested that TFQA can effectively and positively activate neurotransmitters related to cognition and excitability in aging mice. Therefore, TFQA can stimulate neurotrophin signaling to improve synaptic organization and function, as well as enhance neurotransmitter secretion.

### TFQA alleviated neuroinflammation through neurotrophin signaling in aging mice

**Fig. 7:**
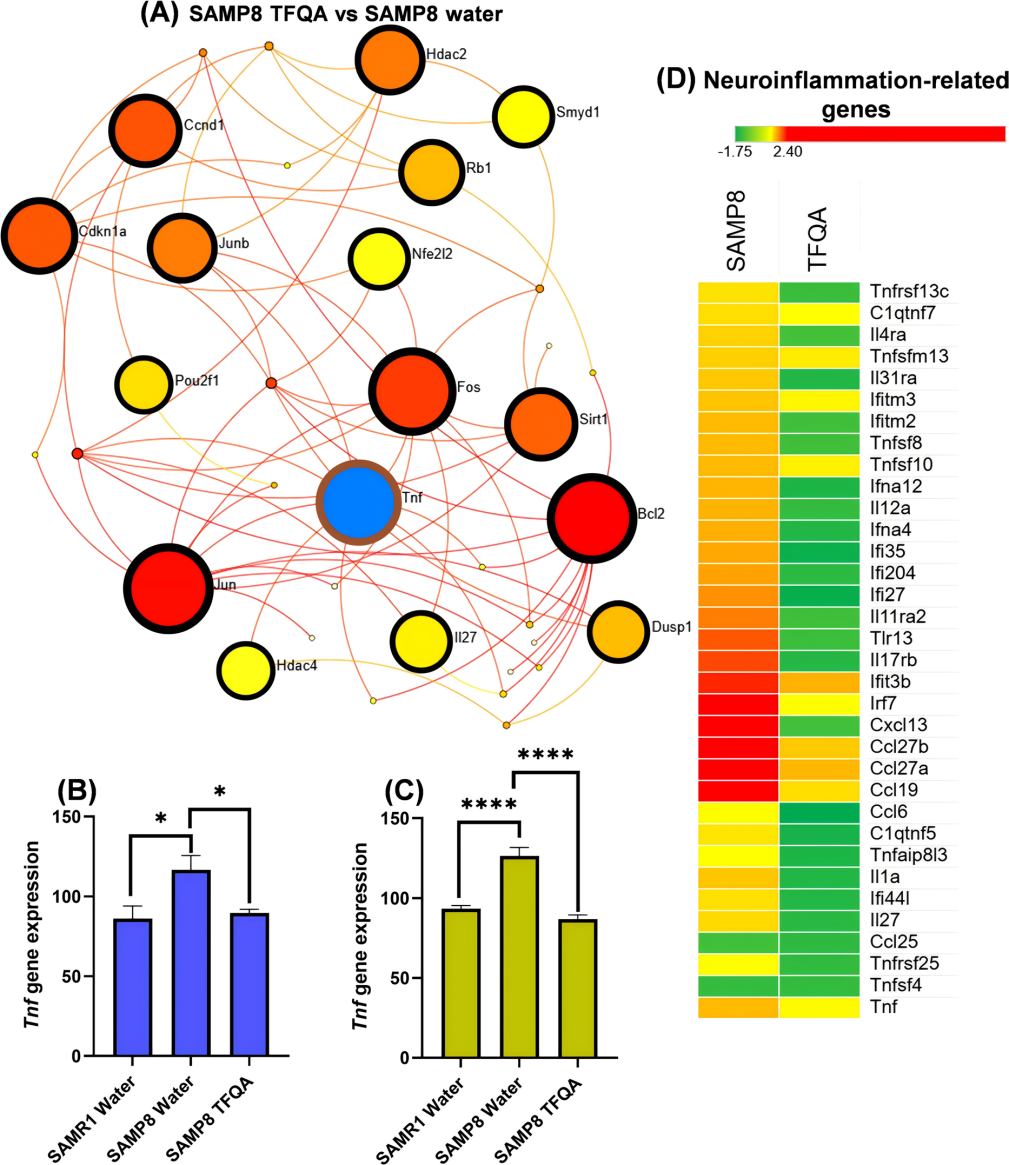
Neuroinflammation-related outcomes in SAMP8 and SAMR1 mice following water or TFQA administration. **A** Molecular interaction network (Module 0) of the neurotrophin signaling pathway in TFQA-treated mice, generated using NetworkAnalyst (https://www.networkanalyst.ca/NetworkAnalyst/home.xhtml). The walktrap algorithm was applied to detect densely connected sub-modules within the global protein–protein interaction (PPI) network. Module 0 represents the largest and most significant cluster. In the network, nodes represent proteins, and edges denote first-order (direct) interactions between them. Seed nodes are a subset of proteins used as starting points for module detection. The color intensity of red in each node reflects the statistical significance of the protein’s involvement in the interaction network (based on fold change). A blue-colored node highlights the most significantly enriched proteins in the neurotrophin signaling pathway network. mRNA expression of *Tnf* measured by quantitative real-time PCR following water and TFQA treatments in SAMP8 and SAMR1 mice. Results are shown for **B** cerebral cortex and **C** hippocampus (*n* = 5 per group). Data are expressed as the mean ± SD of three independent experiments. ****P < 0.0001, ***P < 0.001, **P < 0.01, and *P < 0.05 by ANOVA followed by Tukey’s post hoc test. **D** The heatmap displays fold change (FC) values of neuroinflammation-related genes in SAMP8 (SAMP8 + water vs. SAMR1 + water) and TFQA (SAMP8 + TFQA vs. SAMP8 + water) groups. Red indicates upregulation, and green indicates downregulation

To further explain the anti-aging molecular mechanism of TFQA administration in the neurotrophin signaling pathway, we analyzed the module 0 Walk trap international network of the neurotrophin signaling pathway in TFQA (Fig. 7A). Walk trap works by using random walks to explore the network and identify clusters of proteins that are more densely connected with each other than with other proteins in the network. It is particularly useful for identifying groups of proteins that form functionally coherent sub-networks. As a pro-inflammatory factor, *Tnf* was significantly regulated in the neurotrophin signaling pathway by TFQA. TNF plays a key role in synaptic scaling and neurogenesis. It may also regulate synaptic strength at excitatory synapses by increasing excitatory synaptic transmission and decreasing inhibitory transmission. TNF signaling can trigger a variety of cellular responses, which in turn activate several intracellular signaling pathways, such as NF-κB, p38, Jun N-terminal kinase (JNK), and ceramide/sphingomyelinase, leading to multiple outcomes including inflammation, proliferation, and apoptosis [23].

To further confirm the therapeutic impact of TFQA on pro-inflammatory factors, we analyzed *Tnf* gene expression in the cerebral cortex and hippocampus of the brain. In Fig. 7B and C, compared to SAMR1 water-treated mice and water-treated SAMP8 mice, TFQA-treated aging mice showed significantly decreased *Tnf* expression, with measured values of 116.6 in the cerebral cortex and 126.5 in the hippocampus. In contrast, *Tnf* gene expression in aging mice was 89.5 and 86.9, respectively. It is elucidated that TFQA has a significant effect on inhibiting pro-inflammatory factors in aging. Figure 7D shows the fold changes (FCs) of genes related to neuroinflammation between SAMP8 and TFQA. Compared to SAMP8, there was a marked difference in FCs in TFQA. This further supports the conclusion that TFQA can effectively alleviate neuroinflammation in aging mice. Overall, TFQA reduces *Tnf* expression by activating neurotrophin signaling and alleviating neuroinflammation in aging mice.

### TFQA improved neurotrophic factors in mice brain

**Fig. 8:**
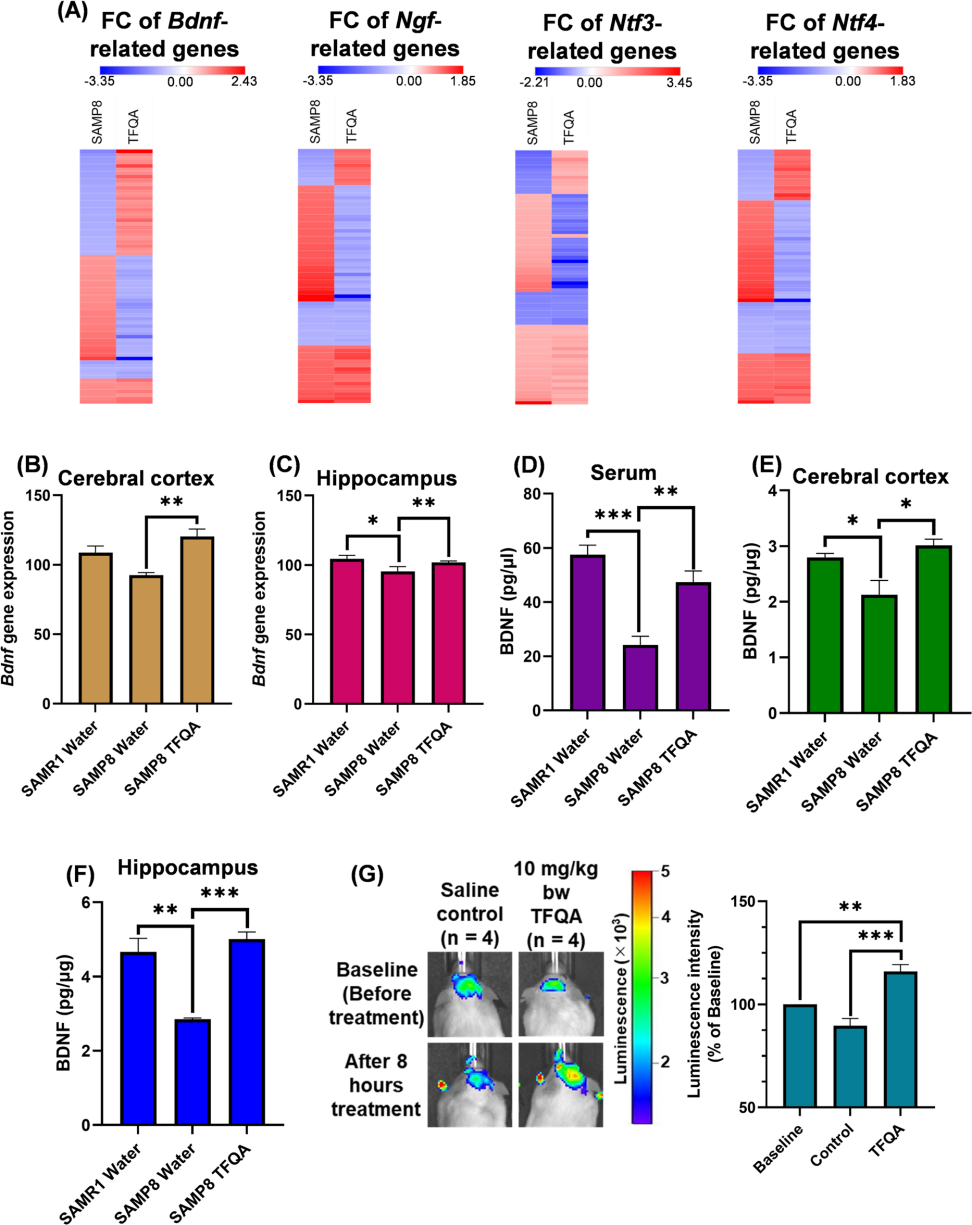
Neurotrophic factor expression following TFQA treatment in SAMP8 mice. **A** The heatmaps are displaying the expression levels (fold change values) of key neurotrophic factors BDNF, NGF, NT-3, and NT-4 in SAMP8 (SAMP8 water vs. SAMR1 water) and TFQA (SAMP8 TFQA vs. SAMP8 water) groups. Red indicates upregulation, and blue indicates downregulation. mRNA expression of *Bdnf* measured by quantitative real-time PCR following water and TFQA treatments in SAMP8 and SAMR1 mice. Results are shown for **B** cerebral cortex and **C** hippocampus (*n* = 3 in each group). Protein levels of BDNF measured by ELISA following water and TFQA treatments in SAMP8 and SAMR1 mice. Results are shown for **D** serum, **E** cerebral cortex, and **F** hippocampus (*n* = 4 in each group). **G** Bioluminescence imaging using AkaLumine-AkaLuc in BDNF-AkaLuc mice before and after 8 h of oral TFQA administration. Representative images show bioluminescence signals, and the rate of increase in luminescence intensity is quantified (*n* = 4 per group). Data are expressed as the mean ± SD of three independent experiments. ****P < 0.0001, ***P < 0.001, **P < 0.01, and *P < 0.05 by ANOVA followed by Tukey’s post hoc test

To explore the target genes in the neurotrophin signaling pathway of TFQA treatment in SAMP8 mice, we analyzed the fold changes (FCs) heat maps of genes related to neurotrophin factors *Bdnf*, *Ngf*, neurotrophin-3 (*Ntf3*), and neurotrophin-4 (*Ntf4*) between SAMP8 and TFQA (Fig. 8A). Compared to the FCs heat maps of genes related to *Ngf*, *Ntf3*, and *Ntf4*, the FCs showed the most differences in *Bdnf*-related genes between SAMP8 and TFQA. Therefore, we further analyzed and verified the regulation of *Bdnf* by TFQA treatment in SAMP8 and SAMR1 mice. In the cerebral cortex and hippocampus, *Bdnf* gene expression increased significantly in SAMP8 mice treated with TFQA (Fig. 8B and C), with values of 120.3 and 101.9, respectively. In the serum, BDNF improved significantly to 47.4 pg/µL in TFQA-treated mice, compared to 24.2 pg/µL in SAMP8 mice treated with water (Fig. 8D). In the hippocampus, BDNF also increased remarkably after TFQA treatment, compared to the aging model (Fig. 8E). The BDNF content of TFQA-treated mice was 5.0 pg/µg, while that of SAMR1 mice was 4.7 pg/µg. In the cerebral cortex, TFQA increased BDNF levels, with a value of 2.1 pg/µg in SAMP8 mice treated with water (Fig. 8F). Additionally, in BDNF-AkaLuc mice, a single oral administration of TFQA significantly augmented the expression of BDNF protein in the mouse brain compared to the saline group (Fig. 8G). These results clarify that TFQA can effectively enhance BDNF to treat aging.

### TFQA exhibited spexcific binding affinity for the BDNF receptor TrkB

**Fig. 9:**
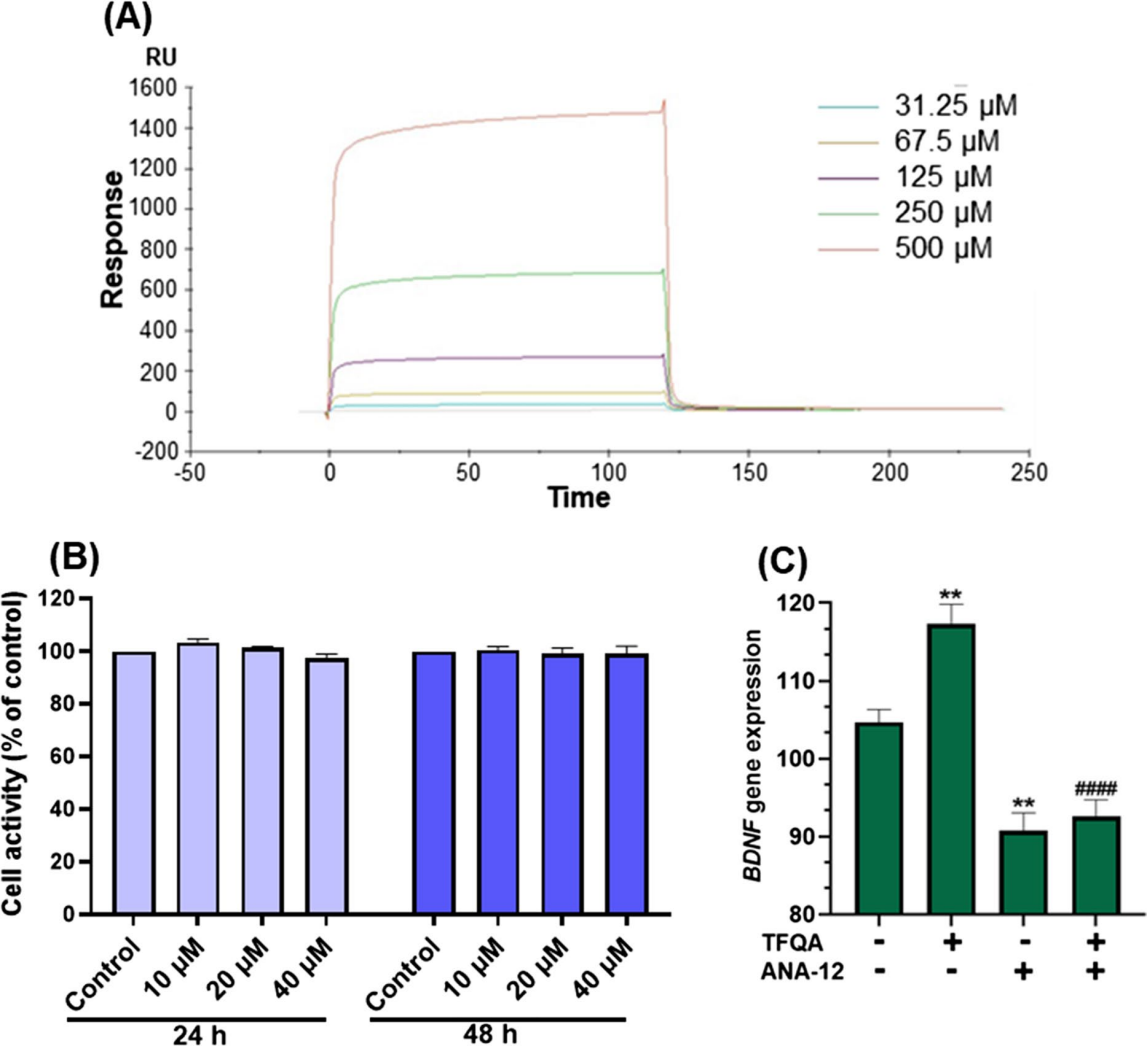
Effects of TFQA on modulation of TrkB, the primary receptor for BDNF. **A** The Biacore assay, a label-free, real-time surface plasmon resonance (SPR) technique, was used to assess the binding affinity of TFQA to TrkB. Binding response values at varying concentrations of TFQA indicate a dose-dependent interaction. **B** Cell activity of SH-SY5Y cells treated with increasing concentrations of TFQA for 24 and 48 h, evaluated using MTT assay (*n* = 4 per group). **C** *BDNF* mRNA expression levels in SH-SY5Y cells following treatment with vehicle control, ANA-12 (a TrkB antagonist), 10 µM TFQA, and 10 µM TFQA combined with ANA-12 for 24 h (*n* = 3 in each group). Data are expressed as the mean ± SD of three independent experiments. *Compared with control, ^#^compared with TFQA-treated group, **P < 0.01 and ^####^*P* < 0.0001 by ANOVA followed by Tukey’s post hoc test

To further support the result of enhanced BDNF in SAMP8 mice treated with TFQA, we also investigated the effect of TFQA treatment on the BDNF receptor, TrkB. As a BDNF receptor, TrkB plays an important role in regulating neurogenesis and repairing neurogenic impairment [24]. We utilized a quantitative method to accurately measure the binding affinities of TFQA to TrkB. The TrkB protein showed stable interactions with TFQA (*Ka* = 2.888 × 10 M^−1^ s^−1^; *K*_*d*_ = 7.207 10^−1^ s^−1^) (Table 1 and Fig. 9A). We found that TFQA bind to the TrkB domain with a *K*_*D*_ of 2.496 10^−2^ M. The obtained dissociation equilibrium constant highlights the relative stability of the TFQA-TrkB complex. The maximum RU value of 1473.522 for TFQA indicates that the surface of TFQA has the capability to bind to TrkB molecules. This demonstrates the significant binding affinity of TFQA for TrkB.

**Table 1:** The response values of TrkB in different concentrations of TFQA

Concentration and compound	Response
31.25 µM TFQA	34.20654
67.5 µM TFQA	92.18701
125 µM TFQA	268.4684
250 µM TFQA	683.1385
500 µM TFQA	1473.522

In SH-SY5Y cells, at 24 and 48 h of treatments, there was no significant difference between the control and TFQA treatment groups (Fig. 9B). TFQA had no significant effect on cell viability and proliferation, and did not show significant toxicity to SH-SY5Y cells. Considering lower concentrations, 10 µM TFQA, along with 24-h treatment time, were selected for further quantitative real-time PCR analysis. In Fig. 9C, *BDNF* expression in 10 µM TFQA-treated groups increased significantly compared to the control group, with values of 117.269. After adding ANA-12, a TrkB antagonist, *BDNF* expression showed a significant decrease in the 10 µM TFQA treatment groups compared to the control group, with values of 92.5471. Overall, TFQA has the potential to specifically target TrkB to stimulate BDNF.

## Discussion

In this study, we conducted a 37-day TFQA intervention in the aging model SAMP8 mice. The results demonstrated that TFQA effectively shortened the escape latency and increased the time spent in the target quadrant and the number of platform crossings, as well as the distance swum and swimming speed. The therapeutic effects of TFQA were explored through a comprehensive analysis of genome, ELISA, and quantitative real-time PCR. Findings revealed that TFQA ameliorates neuroinflammation, improves neurotransmitter secretion, and enhances synaptic organization by enriching the neurotrophin signaling pathway and boosting the gene expression and protein content of BDNF in aging mice. Moreover, in BDNF-AkaLuc mice, TFQA administration significantly enhanced the expression of BDNF protein in the mouse brain, further confirming that TFQA can remarkably stimulate BDNF translation. In the biacore assay, a potential affinity between TFQA and the BDNF receptor TrkB was observed. In addition, in SH-SY5Y cells, TFQA treatment not only increased the gene expression of *BDNF* but also inhibited the effect of TFQA on increasing BDNF when the TrkB inhibitor ANA-12 was added. TFQA may specifically enhance binding with TrkB.

During normal aging, various memory, cognitive, and motor-related processes gradually undergo process-specific decline [3]. The SAMP8 mouse strain serves as an established aging model characterized by premature onset of learning and memory impairments. Notably, pharmacological interventions demonstrate age-dependent efficacy in improving memory processing functions in these P8 mice [25]. The MWM test was introduced two decades ago as a tool for investigating spatial learning and memory in laboratory mice. Since then, it has become one of the most widely used experimental paradigms in behavioral neuroscience [26]. Firstly, we investigated the cognitive ability through the MWM test after TFQA administration. These results align with existing research on natural compounds and pharmacological treatments, confirming that SAMP8 mice display significant deficits in cognitive function and spatial learning as evidenced by their impaired performance in the MWM test [27, 28]. Following treatment administration, we observed comprehensive improvements in multiple behavioral metrics: the escape latency during training trials was significantly shortened, total swimming duration decreased while swimming speed increased, the number of platform crossings rose substantially, and the time spent in the target quadrant showed notable prolongation. These quantitative behavioral improvements collectively demonstrate the therapeutic efficacy of the intervention in ameliorating age-related cognitive decline in this established murine model of accelerated aging.

Our GO (Gene Ontology) analysis revealed significant enrichment of synaptic transmission, neurotransmitter secretion, and calcium ion transport processes in TFQA-treated SAMP8 mice (Fig. 4). The aging nervous system undergoes progressive functional decline across multiple cognitive domains, mediated through complex neurobiological mechanisms. Current neurogerontological research demonstrates that region-specific neuronal vulnerability and synaptic loss disrupt functional neural networks. At the cellular level, age-related neurodegeneration manifests as (1) synaptic density reduction and plasticity impairment, (2) neurotransmitter receptor dysregulation, (3) neurochemical signaling imbalances, (4) cytoskeletal reorganization and organelle dysfunction, and (5) compromised action potential propagation. These multiscale alterations collectively underlie the pathogenesis of age-associated cognitive impairment [29]. Accumulating evidence indicates that disruption of these synaptic connections contributes significantly to age-related brain alterations and cognitive decline [30]. Advanced in vivo two-photon imaging studies in aged mice have revealed impaired basal synaptic dynamics in cortical dendrites and presynaptic terminals, accompanied by a marked reduction in learning-induced formation of new dendritic spines [31]. Notably, quantitative ultrastructural analyses demonstrate a significant decrease in synaptic vesicle density at hippocampal mossy fiber-CA3 and CA3-CA1 synaptic terminals during aging [32]. These age-dependent structural synaptic modifications are functionally consequential, leading to measurable deficits in neural circuit operation and cognitive performance. Consistent findings across multiple studies have established that aging is associated with characteristic alterations in synaptic transmission, excitability, and plasticity within hippocampal and cortical networks. However, our current understanding remains limited regarding the underlying mechanisms driving these alterations, as well as the precise relationship between TFQA and age-associated synaptic remodeling and cognitive deterioration. Advanced aging appears to be associated with progressive dysregulation across multiple neurotransmitter systems that collectively modulate brain function. The observed decline in neurotransmitter biosynthesis capacity may be mechanistically linked to age-related perturbations in circulating metabolites. Particularly during advanced aging phases, cumulative neuropathological processes—including selective neuronal vulnerability, age-associated pathologies, and other comorbid conditions—may synergistically exacerbate alterations in central neurotransmitter dynamics [33]. Regarding the enhanced calcium ion transport processes induced by TFQA administration, current evidence suggests that this compound may improve cerebral calcium homeostasis through dual mechanisms: mitigating oxidative damage to calmodulin (thereby restoring plasma membrane Ca^2+^-ATPase activation) and directly enhancing plasma membrane Ca^2+^-ATPase functionality. These synergistic effects collectively improve neuronal calcium clearance capacity and consequently ameliorate cognitive performance [34].

In the KEGG result of this research, TFQA treatment significantly regulated the neurotrophin signaling (Fig. 5). Neurotrophic factors represent a family of secreted proteins indispensable for neuronal development. Extensive studies have established NGF as crucial for the survival and development of the peripheral nervous system [35]. Importantly, NGF undergoes retrograde transport from peripheral target sites to neuronal cell bodies, thereby promoting survival during developmental programmed cell death [36]. In the central nervous system, BDNF has been identified as the predominant neurotrophic factor, where its abundant expression modulates multiple aspects of neuronal function including growth, morphological differentiation, synaptic plasticity, and structural remodeling [7]. In Fig. 6A, B, C, and D and Table S7, S8, S9, and S10, the hub node lists of the neurotrophin signaling pathway in TFQA remarkably regulated the synaptic functions, specially the presynaptic and postsynaptic functions. Evidence suggests that neurotrophins can modulate synaptic transmission through both presynaptic and postsynaptic mechanisms. The neuronal study provided direct evidence supporting the presynaptic action site, where adenovirus-mediated expression of truncated TrkB receptors in presynaptic neurons abolished BDNF-induced potentiation of both evoked and spontaneous synaptic transmission [37]. On the other hand, substantial evidence demonstrates that neurotrophic factors exert significant postsynaptic effects. Microinjection of receptor tyrosine kinase inhibitors into postsynaptically cultured hippocampal neurons effectively suppresses neurotrophin-induced increases in firing frequency and the accompanying enhancement of synaptic currents [38]. At postsynaptic sites, BDNF application potentiates N-methyl-D-aspartate (NMDA) receptor channel activity in both cortical and hippocampal cultures, while NMDA receptor antagonists completely block BDNF-induced augmentation of spontaneous synaptic currents [39]. Furthermore, in hippocampal slices, BDNF-mediated synaptic potentiation requires putative postsynaptic protein synthesis [40]. These collective findings underscore the critical need to precisely delineate the subcellular localization of neurotrophic factor actions (presynaptic versus postsynaptic) and identify their coupled signaling components.

TFQA treatment significantly increased the levels of key neurotransmitters including serotonin, norepinephrine, and dopamine, demonstrating its potential neuromodulatory effects (Fig. 6E–G). Firstly, the loss of serotonergic neurons and neurotransmitters in normal aging and late-life neuropsychiatric disorders may contribute to behavioral changes commonly observed in the aging population [41]. Serotonin depletion impairs memory formation, while enhanced serotonergic function promotes mnemonic processes. With respect to learning and memory, a key functional consequence of elevated serotonin levels is the modulation of more direct neurophysiological effects mediated by other neurotransmitter systems [42] and neurotrophic factors [43]. Furthermore, noradrenergic activity shows selective engagement in the cerebral cortex during spatial working memory tasks [44]. The pharmacological damage induced by 1-methyl-4-phenyl-1,2,3,6-tetrahydropyridine (MPTP) leads to noradrenergic dysfunction, which is associated with impairments in attention, attentional shifting, working memory, cognitive flexibility, and problem-solving abilities [45]. Furthermore, neurotrophic factors promote the survival of noradrenergic neurons and upregulate norepinephrine uptake. These factors are considered crucial for maintaining noradrenergic innervation in the aging brain [46]. This suggests that stimulating noradrenergic activity through engaging activities could be an effective approach for preventing neurodegenerative diseases throughout life [47]. Regarding dopamine, with aging, the decline in dopamine production and receptor sensitivity contributes to cognitive slowing, motor impairments, and mood disorders. The increase in dopamine is crucial for treating both age-related changes in brain function and neurodegenerative diseases [48]. Impairment of dopaminergic pathways induces cognitive deficits across multiple domains in rodents and primates, including memory, attention, and inhibitory control. Human studies demonstrate that dopaminergic agonists may enhance performance, whereas antagonists impair performance in specific executive function and working memory tasks [49]. During aging, BDNF knockout mice exhibit reductions in both dopaminergic neuron size and motor coordination [50]. These findings suggest that TFQA may enhance synaptic plasticity and improve cognitive capabilities by promoting neurotransmitter release through activation of neurotrophic factors. During learning processes, neural inputs activate cerebral immune cells—including T cells and microglia—via neurotransmission and neurohormonal pathways. These activated immune cells secrete diverse mediators such as inflammatory cytokines and neurotrophic factors, which collectively facilitate plasticity-associated processes in neurons, astrocytes, and neural precursor cells. Such neuroimmune interactions ultimately consolidate spatial memory and hippocampal function while promoting neurogenesis [51]. This was confirmed by our analysis of T neuroinflammation in TFQA-treated aging mice (Fig. 7). For the pro-inflammatory factor *Tnf*, after TFQA treatment, *Tnf* expression was significantly decreased in the cerebral cortex and hippocampus. TFQA treatment significantly reduced TNF expression in both the cerebral cortex and hippocampus, contrasting with its marked elevation in elderly populations that establishes TNF as a validated biomarker of age-related chronic inflammation [52]. As a key mediator of neuroinflammation-induced neuronal dysfunction and cognitive impairment, TNF’s pathogenic role is further demonstrated by the ability of its synthesis inhibitors to reverse hippocampal deficits [53], while the flavonoid naringenin ameliorates age-related neuronal loss and improves spatial memory in aged mice through plasma TNF downregulation [54]. Notably, BDNF exerts neuroprotection by suppressing lipopolysaccharide-induced TNF production, with TNF’s neurotoxic effects being attenuated through TrkB receptor blockade [55]. These collective findings provide compelling evidence that upregulating neurotrophic signaling pathways offers a promising therapeutic approach for combating neuroinflammation during aging.

In the BDNF experimental results, TFQA demonstrated significant binding affinity for TrkB (the receptor for BDNF) and markedly enhanced both *Bdnf* gene expression and BDNF protein levels (Fig. 8). Emerging evidence highlights TrkB-targeted pharmacological strategies for neurotrophic modulation, where various compounds exert neuroprotective effects through TrkB affinity. Specifically, the natural compound oleacein functions as a TrkB agonist, demonstrating potential anti-neuroinflammatory and antidepressant properties via BDNF/TrkB pathway modulation [56]. Clinically, rapid-acting antidepressants like fluoxetine directly bind to the transmembrane domain of TrkB, facilitating its synaptic localization and subsequent BDNF activation, thereby enhancing neurite outgrowth and dendritic arborization [57]. These findings provide compelling evidence that enhancing the binding affinity between TFQA and TrkB effectively promotes synaptic growth. The elevated BDNF levels induced by TFQA administration exhibit high specificity in activating TrkB receptors, thereby enhancing neuronal survival, differentiation, and synaptic function. BDNF has demonstrated neuroprotective efficacy by activating TrkB in both the hippocampus and striatum of mice, while also improving motor learning capacity in rats following traumatic brain injury [58]. Notably, age-related impairments in GABAergic neuroplasticity resulting from BDNF mRNA knockdown in aged mice can be effectively rescued through prolonged exogenous BDNF treatment, leading to significant cognitive improvement [59]. Although TFQA administration in aging mice has demonstrated anti-aging effects by upregulating neurotrophic signaling (particularly BDNF) and stimulating synaptic and neurotransmitter activity, certain aspects of its underlying mechanisms require further clarification. Specifically, whether TFQA acts directly on neurotrophic pathways in the brain or through secondary mediators remains to be fully determined, as do the detailed molecular processes linking neurotrophic signaling to enhanced synaptic growth and function.

While our study establishes the anti-aging effects of TFQA on enhancing neurotrophin signaling, several considerations merit consideration. Given the synthetic nature of TFQA, future studies should prioritize two key translational directions: (1) systematic evaluation of its pharmacokinetic profile and blood–brain barrier penetration to optimize dosing regimens, and (2) preclinical validation in non-human primate models of age-related cognitive decline to bridge the gap toward human trials. Additionally, structure–activity relationship studies could further refine TFQA’s selectivity for TrkB over related tyrosine kinase receptors, minimizing off-target effects. These efforts would strengthen its potential as a clinically viable neurotrophic modulator, particularly for neurodegenerative conditions where endogenous BDNF signaling is compromised.

## Conclusions

The development of new strategies to promote nervous system development in aging-related diseases is a major therapeutic challenge. Our results suggest that in aging SAMP8 mice, TFQA treatment improves spatial learning and memory through increased neurotransmitter secretion, enhanced synaptic organization, and the amelioration of neuroinflammation by significantly stimulating BDNF in the neurotrophin signaling pathway. Interestingly, we also found that TFQA has a special effect on promoting *BDNF* in SH-SY5Y cells, and there is the potential affinity between TFQA and TrkB. Moreover, it is important to determine how TFQA binds to TrkB to enhance biological activity. Together, we conclude that TFQA is a promising novel drug for improving spatial memory, cognition, and treating aging.

## Supplementary Information

Below is the link to the electronic supplementary material.

ESM 1 (DOCX 39.6 KB)

## Data Availability

Microarray dataset is deposited in Gene Expression Omnibus (GEO) under Accession Number: GSE283354.

## Abbreviations

*AD*: Alzheimer’s disease
*PD*: Parkinson’s disease
*Bdnf*: Brain-derived neurotrophic factor
*TrkB*: Tyrosine receptor kinase B
*QA*: Quinic acid
*Tnf*: Tumor necrosis factor
*NGF*: Nerve growth factor
*FQA*: Feruloylquinic acid
*ERK*: Extracellular signal-regulated kinase
*TFQA*: 3,4,5-Tri-Feruloylquinic acid
*SAMP8*: Senescence-accelerated mouse prone 8
*SAMR1*: Senescence-accelerated mouse resistant 1
*MWM*: Morris water maze
*DAVID*: Database for Annotation, Visualization, and Integrated Discovery
*SynGO*: Synaptic Gene Ontologies
*ELISA*: Enzyme-linked immuno-sorbent assay
*AkaLuc*: Aequorin-luciferase reporter
*MTT*: 3-(4,5-Dimethylthiazol-2-yl)-2,5-diphenyltetrazolium bromide
*DEG*: Differentially expressed gene
*FC*: Fold change
*BP*: Biological processes
*CC*: Cellular components
*GO*: Gene Ontology
*Wnt*: Wingless-related integration site
*PPI*: Protein-protein interaction
*KEGG*: Kyoto Encyclopedia of Genes and Genomes
*PI3K-Akt*: Phosphatidylinositol 3-kinase/protein kinase B
*MAPK*: Mitogen-activated protein kinase
*mTOR*: Mammalian target of rapamycin
*NF-κB*: Nuclear component kappa B
*JNK*: Jun N-terminal kinase
*Aβ*: Amyloid beta peptide
*NMDA*: N-Methyl-D-aspartate
*MPTP*: 1-Methyl-4-phenyl-1,2,3,6-tetrahydropyridine
