# Supplementary material for: A novel synthetic 3,4,5-tri-feruloylquinic acid enhances learning and memory via neurotrophin signaling in an aging model senescence-accelerated prone 8 mice
Source: GeroScience. 2025 Jul 23;48(2):2563–86. doi: 10.1007/s11357-025-01783-7 (PMC12972279; doi:10.1007/s11357-025-01783-7)
Supplement: Supplementary file 1 — (DOCX 39.6 KB) [file 11357_2025_1783_MOESM1_ESM.docx]

SUPPORTING INFORMATION

A novel synthetic 3,4,5-tri-Feruloylquinic acid enhances learning and memory via neurotrophin signaling in an aging model senescence-accelerated prone 8 mice

*Hongyu Lin^1^, Kazunori Sasaki^2, 3^, Farhana Ferdousi**^1,3, 4^,* *Shinji Kondo^3^*, *Hiroko Isoda^1, 2, 3, 4, *^*

1. Tsukuba Life Science Innovation (T-LSI) Program, University of Tsukuba, Tsukuba 305-8577, Japan
2. Open Innovation Laboratory for Food and Medicinal Resource Engineering (FoodMed-OIL), National Institute of Advanced Industrial Science and Technology (AIST), Tsukuba 305-8577, Japan
3. Alliance for Research on the Mediterranean and North Africa (ARENA), University of Tsukuba, Tsukuba 305-8577, Japan
4. Institute of Life and Environmental Sciences, University of Tsukuba, Tsukuba 305-8572, Japan

**Content**

[**Table S1.** Data of escape latency (s) in SAMP8 and SAMR1 mice with TFQA and water treatments 3](#_Toc197464462)

[**Table S2.** Data of time spent in the target quadrant, numbers of crossings of platform, distance and speed of swimming during learning in SAMP8 and SAMR1 mice with TFQA and water treatments 4](#_Toc197464463)

[**Table S3.** Summary of BP and CC enriched by downregulated DEGs in SAMP8 water vs SAMR1 water 5](#_Toc197464464)

[**Table S4.** Summary of BP enriched by upregulated DEGs in SAMP8 water vs SAMR1 water 6](#_Toc197464465)

[**Table S5.** Summary of BP and CC enriched by upregulated DEGs in SAMP8 TFQA vs SAMP8 water 7](#_Toc197464466)

[**Table S6.** Summary of BP enriched by downregulated DEGs in SAMP8 TFQA vs SAMP8 water 8](#_Toc197464467)

[**Table S7.** Summary of BP terms from the hub genes of neurotrophin signaling pathway of SAMP8 9](#_Toc197464468)

[**Table S8.** Summary of CC terms from the hub genes of neurotrophin signaling pathway of SAMP8 10](#_Toc197464469)

[**Table S9.** Summary of BP terms from the hub genes of neurotrophin signaling pathway of TFQA 11](#_Toc197464470)

[**Table S10.** Summary of CC terms from the hub genes of neurotrophin signaling pathway of TFQA 12](#_Toc197464471)

**Table S1.** Data of escape latency (s) in SAMP8 and SAMR1 mice with TFQA and water treatments

|  | **Day 1** | **Day 2** | **Day 3** | **Day 4** | **Day 5** | **Day 6** | **Day 7** |
| --- | --- | --- | --- | --- | --- | --- | --- |
| **SAMR1 water** | 37.08 | 36.12 | 37.90 | 30.68 | 34.68 | 29.55 | 21.44 |
| **SAMP8 water** | 56.78 | 48.88 | 55.05 | 48.58 | 46.72 | 51.88 | 47.81 |
| **SAMP8 TFQA** | 54.60 | 46.47 | 35.99 | 36.87 | 29.75 | 31.73 | 22.05 |

**Table S2.** Data of time spent in the target quadrant, numbers of crossings of platform, distance and speed of swimming during learning in SAMP8 and SAMR1 mice with TFQA and water treatments

|  | **Time (s)** | **Numbers** | **Distance (cm)** | **Speed (cm/s)** |
| --- | --- | --- | --- | --- |
| **SAMR1 water** | 20.42 | 1.67 | 11403.37 | 180.16 |
| **SAMP8 water** | 21.03 | 0.17 | 5890.63 | 90.32 |
| **SAMP8 TFQA** | 28.14 | 1.75 | 7877.93 | 127.46 |

**Table S3.** Summary of BP and CC enriched by downregulated DEGs in SAMP8 water vs SAMR1 water

| **ID** | **Term** | **Enrichment** | **Count** | **Class** |
| --- | --- | --- | --- | --- |
| GO:0048814 | Regulation of dendrite morphogenesis | 7.41 | 3 | BP |
| GO:0046928 | Regulation of neurotransmitter secretion | 7.14 | 3 | BP |
| GO:0007269 | Neurotransmitter secretion | 5.84 | 3 | BP |
| GO:1990090 | Cellular response to nerve growth factor stimulus | 5.71 | 4 | BP |
| GO:0007218 | Neuropeptide signaling pathway | 5.59 | 8 | BP |
| GO:0007049 | Cell cycle | 1.58 | 16 | BP |
| GO:0030672 | Synaptic vesicle membrane | 4.13 | 10 | CC |
| GO:0098982 | GABA-ergic synapse | 3.81 | 10 | CC |
| GO:0043025 | Neuronal cell body | 1.99 | 21 | CC |
| GO:0043005 | Neuron projection | 1.92 | 12 | CC |
| GO:0045202 | Synapse | 1.72 | 28 | CC |
| GO:0098978 | Glutamatergic synapse | 1.66 | 21 | CC |

**Table S4.** Summary of BP enriched by upregulated DEGs in SAMP8 water vs SAMR1 water

| **ID** | **Term** | **Enrichment** | **Count** | **Class** |
| --- | --- | --- | --- | --- |
| GO:0033198 | Response to ATP | 5.05 | 4 | BP |
| GO:0045577 | Regulation of B cell differentiation | 12.96 | 3 | BP |
| GO:0060337 | Type I interferon-mediated signaling pathway | 7.56 | 4 | BP |
| GO:0071346 | Cellular response to type II interferon | 5.41 | 11 | BP |
| GO:0035458 | Cellular response to interferon-beta | 5.04 | 6 | BP |
| GO:0033077 | T cell differentiation in thymus | 5.04 | 4 | BP |
| GO:0002376 | Immune system process | 2.94 | 27 | BP |
| GO:0006915 | Apoptotic process | 1.84 | 20 | BP |

**Table S5.** Summary of BP and CC enriched by upregulated DEGs in SAMP8 TFQA vs SAMP8 water

| **ID** | **Term** | **Enrichment** | **Count** | **Class** |
| --- | --- | --- | --- | --- |
| GO:0007269 | Neurotransmitter secretion | 6.30 | 4 | BP |
| GO:0010975 | Regulation of neuron projection development | 4.83 | 4 | BP |
| GO:0007268 | Chemical synaptic transmission | 3.76 | 12 | BP |
| GO:0050808 | Synapse organization | 3.75 | 7 | BP |
| GO:0035249 | Synaptic transmission, glutamatergic | 3.65 | 4 | BP |
| GO:0071805 | Potassium ion transmembrane transport | 3.63 | 9 | BP |
| GO:0007612 | Learning | 3.42 | 5 | BP |
| GO:0006816 | Calcium ion transport | 2.77 | 8 | BP |
| GO:0007420 | Brain development | 2.33 | 11 | BP |
| GO:0007049 | Cell cycle | 1.92 | 24 | BP |
| GO:0030154 | Cell differentiation | 1.55 | 30 | BP |
| GO:0060076 | Excitatory synapse | 5.33 | 5 | CC |
| GO:0043025 | Neuronal cell body | 2.88 | 38 | CC |
| GO:0098982 | GABA-ergic synapse | 2.74 | 9 | CC |
| GO:0043005 | Neuron projection | 2.69 | 21 | CC |
| GO:0098978 | Glutamatergic synapse | 2.47 | 39 | CC |
| GO:0031982 | Vesicle | 2.25 | 8 | CC |
| GO:0045202 | Synapse | 2.60 | 42 | CC |

**Table S6.** Summary of BP enriched by downregulated DEGs in SAMP8 TFQA vs SAMP8 water

| **ID** | **Term** | **Enrichment** | **Count** | **Class** |
| --- | --- | --- | --- | --- |
| GO:0016055 | Wnt signaling pathway | 2.03 | 10 | BP |
| GO:0006954 | Inflammatory response | 2.01 | 17 | BP |
| GO:0002376 | Immune system process | 1.75 | 20 | BP |
| GO:0006915 | Apoptotic process | 1.70 | 23 | BP |

**Table S7.** Summary of BP terms from the hub genes of neurotrophin signaling pathway of SAMP8

| **BP ontology terms** | **Gene count** | **p-value** | **q-value** |
| --- | --- | --- | --- |
| process in the synapse | 5 | 0.2423 | 0.2423 |
| synapse organization | 3 | 0.1349 | 0.2423 |

**Table S8.** Summary of CC terms from the hub genes of neurotrophin signaling pathway of SAMP8

| **CC ontology terms** | **Gene count** | **p-value** | **q-value** |
| --- | --- | --- | --- |
| postsynapse | 5 | 0.1369 | 0.2738 |
| synapse | 6 | 0.2807 | 0.2807 |

**Table S9.** Summary of BP terms from the hub genes of neurotrophin signaling pathway of TFQA

| **BP ontology terms** | **Gene count** | **p-value** | **q-value** |
| --- | --- | --- | --- |
| process in the postsynapse | 7 | 4.68e-5 | 3.28e-4 |
| regulation of postsynaptic membrane neurotransmitter receptor levels | 5 | 3.06e-4 | 1.07e-3 |
| process in the synapse | 11 | 1.42e-3 | 2.48e-3 |
| neurotransmitter receptor localization to postsynaptic specialization membrane | 3 | 1.20e-3 | 2.48e-3 |
| synapse organization | 6 | 3.48e-3 | 4.87e-3 |
| synapse assembly | 3 | 0.0193 | 0.0225 |
| chemical synaptic transmission | 3 | 0.0249 | 0.0249 |

**Table S10.** Summary of CC terms from the hub genes of neurotrophin signaling pathway of TFQA

| **CC ontology terms** | **Gene count** | **p-value** | **q-value** |
| --- | --- | --- | --- |
| postsynaptic density | 9 | 3.30e-6 | 1.98e-5 |
| postsynapse | 12 | 5.88e-5 | 1.76e-4 |
| synapse | 15 | 1.18e-4 | 2.36e-4 |
| integral component of postsynaptic density membrane | 3 | 7.37e-3 | 0.0111 |
| integral component of presynaptic membrane | 3 | 0.0107 | 0.0128 |
| presynapse | 6 | 0.0319 | 0.0319 |
